# The concepts of “domestication” and “artificial selection” and their significance in the work of Charles Darwin

**DOI:** 10.1590/S0104-59702026000100033en

**Published:** 2026-08-14

**Authors:** Miriam Rebeca Alvarez Tostado Reyes, Fabiola Juárez-Barrera, Alfredo Bueno-Hernández

**Affiliations:** i Coordination of Graduate Studies, Graduate Program in Biological Sciences, Universidad Nacional Autónoma de México. Mexico City – Mexico. mirirbk29@gmail.com; ii Professor, Faculty of Higher Studies Zaragoza, Universidad Nacional Autónoma de México. Mexico City – Mexico. fabiolajuarezunam@gmail.com; iii In memoriam

**Keywords:** Domestication, Artificial selection, Hybridization, Crossing, Close interbreeding

## Abstract

This analysis examines the terms “domestication” and “selection” presented in Darwin’s *The variation of animals and plants under domestication*, and the significance he gave to these concepts. This approach focuses on how Darwin drew upon knowledge generated centuries earlier by experts in breeding and cultivation to modify prevailing definitions and use this semantic shift as a primary instrument to defend his theory. The construction of the “analogy” between natural and artificial selection was not merely a heuristic resource, but rather the result of an inductive generalization grounded in irrefutable evidence. Both concepts were reinterpreted based on Darwinian transmutationism, as specific evolutionary scenarios.

The evolutionary connotation of “domestication” dates back to the 19th century, a period in which the debate between the Fixist doctrine and the transmutationist vision of species emerged and developed. This new meaning originated particularly in the Darwinian work, even though before Darwin, other naturalists had considered this process as evidence of the mutability of species (Burkhardt, 1995; Corsi, 2005; Galera, 2016; Friedman, Endress, 2020). Some authors perceived domesticated forms as artificial productions that could be reversed, thus not transcending the essentially fixed character of the species category (Corsi, 2004, p.224, 2012, p.31).

For Darwin, domestication represented not only a model of the tendency toward gradual modification but a transformative process in itself. This work investigates two main questions: What significance did Darwin attribute to the notions of “domestication” and “artificial selection”? What was the importance of these concepts within his theory?

Examining the redefinition proposed by Darwin is important from various perspectives. First, our historical analysis helps us understand that, contrary to the views of Coppinger and Smith (1983), Darwin did not see the process of domestication as separate from nature or merely as a metaphor for the species evolution (Richards, 1998; Ritvo, 2013). Additionally, our contribution is relevant because it contextualizes how Darwin used the concept of “domestication” in some of his most significant scientific works, representing the origin of the evolutionary aspect of this term. Although the role of domestication and artificial selection in Darwinian thought has been extensively explored (Ruse, 1975; Evans, 1984; Bartley, 1992; Browne, 2008; Varno, 2011; Hodge, 2012; Theunissen, 2012), our approach focuses on how Darwin drew upon facts derived from practical breeding and cultivation experience to modify the meaning of these existing concepts. This semantic shift became a crucial tool in defending his theory. The new meaning he assigned to “domestication” and “artificial selection” helped to frame the mechanisms involved in the development of domesticated breeds as universal processes, processes that also apply to species found in the wild.

A historical analysis of these components, contained within his work, will allow for a broader and more nuanced understanding of how he identified an innovative significance, and an appreciation of the importance of these components within his theory. These elements provided sufficient evidence that varieties tend to diverge from their original type in response to their environment, and that variation, as a universal attribute, represented a potentially limitless tendency.

Before the development of evolutionary theory, the existence of selective conditions affecting managed resources was recognized. These conditions resembled the pressures experienced by organisms in the wild, though they differed in terms of temporal scale. Significant advancements in the concept of “selection” occurred throughout various regions of Europe in the late 18th century, even before the arrival of Darwinian thought. These developments were rooted in the practices of farmers and breeders (Theunissen, 2012; Hodge, 2012; Derry, 2015; Poczai, Santiago-Blay, 2021). A notable example of this emerged in Central Europe, particularly in Moravia (present-day Czech Republic). There, sheep breeders organized in private societies dedicated to discussing improvements in livestock breeding techniques. Within these circles, the concept of *künstliche Zuchtwahl*, which translates to “artificial selection,” emerged. Proposed by Christian Carl André in 1812, the term referred to the modification of animal forms and characteristics through specific breeding techniques, which were understood to have generated the known domestic breeds (Poczai, Santiago-Blay, 2022, p.11). The explanations derived from records, exchanges, and experimentation regarding the improvement of breeds and varieties frequently extended into scientific debates and theories, as the effects of selection on domesticated organisms provided essential information regarding patterns of variation and heredity.

Even though Charles Darwin was not the only naturalist who paid attention to domesticated species to support explanations about the mutability of species, it is possible to say that he was one of the first authors to consider the process of domestication as an experimental practice demonstrating the modification and succession of species as a result of the concatenation of certain components: variability, heredity, and differential survival-reproduction.

The underlying premise of this study is that reviewing publications and texts focused on the practices and theories of domestication, and their significance, during the 19th century will provide a historical perspective on this discipline. This will allow for an analysis of its relationship with evolutionary theory as it became integrated into the new paradigm. Charles Darwin’s work *The variation of animals and plants under domestication* (1868) is useful for this purpose for two reasons: (1) before its publication, the debate regarding the evolution of species and its underlying mechanisms was already part of the European intellectual climate; this began in 1809 with the publication of Jean-Baptiste Lamarck’s *Philosophie zoologique*, and throughout the subsequent five decades, various works had been published specifically to address the general concept of the transmutation of species (Corsi, 2005, p.68; Galera, 2017, p.29); (2) in *Variation*, Darwin (1868) presented extensive experimental and empirical evidence drawn from various authors. Some of these authors were engaged in livestock breeding or agriculture, while others were naturalists interested in the mechanisms of variation, heredity, reproduction, and modification. Consequently, his research offers a representative sample of the specialized techniques and theoretical developments surrounding domestication during this transitional period. Through the Darwinian interpretation, the scientific study of domestication enabled the conceptualization of this process as a model for observing and understanding changes in living organisms. This is predicated on the idea that the components essential to domestication are, in essence, the very same elements required for the mutability of species to occur (Varno, 2011, p.42).

Since domestication involves the selection of traits in individuals, it is common to assume that “domestication” and “artificial selection” may be equivalent terms. *Variation* was one of the first publications to address the relationship between these processes and interpret them as evolutionary models. Although they are undoubtedly closely related concepts, our exploration reveals that, while Darwin did not explicitly articulate a distinction between the two, he consistently used a recognizably different usage for each term.

We will start examining the significance of the general concept of selection as it emerged from experts in breeding and cultivation, and how Darwin interpreted this concept when applying it to his notion of artificial selection. We will also analyze the role of selective breeding practices, such as hybridization, crossing, and close interbreeding, as the most robust body of evidence Darwin used to explain the fundamental role of variation in the morphological differentiation among varieties. The generalizations drawn from these observations enabled him to extend his argument to wild environments. Finally, we will discuss Darwin’s interpretation of the concept of “domestication” as a distinct evolutionary process, demonstrating that certain prevailing beliefs, such as the notions that variation always had a fixed limit, that it was reversible, or that varieties could never transcend the species boundary, were conceptions contradicted by the empirical facts. The mutability of living organisms was manifested across all types of scenarios, even when the context was constrained by specific conditions, such as human intervention and the temporal scale involved.

## The principle of “selection” in Darwinian thought

Since an early stage, shortly after his return from the journey on the HMS Beagle, domestication had an important place in Darwin’s transformist reflections (Ruse, 1975; Evans, 1984; Bartley, 1992; Varno, 2011; Theunissen, 2012). He began to carefully examine this clue for the construction of his theory in his notebooks, particularly in volume C (February-July 1838) (Ruse, 1975, p.344; Evans, 1984, p.122). In 1840, following the reading of *Cattle, their breeds, management, and disease* by William Youatt (1834), Darwin started to use the terms *picking* and *selection* to refer to the nature of varieties and their relationship to the improvement of plants and animals (Evans, 1984).

The general concept of “selection” was present in the specialized literature on selective breeding dating back to the 18th century (Wood, 2003, p.27; Poczai, Santiago-Blay, 2021, p.5, 2022, p.6). This idea highlighted the need to choose, or harvest, the most remarkable, productive, or distinctive individuals, as an almost invariable outcome was expected, attributed to the ancient adage that “like begets like” (Wilkinson, 1820, p.20; Youatt, 1834, p.60). The 19th century witnessed a further expansion of perspectives regarding the theory of selection and its manifold applications. Administrative structures were established for cultivation and breeding – whether through government interventions aimed at developing the agricultural sectors (Macdonald, 2017, p.21), or through the organization of private individuals into livestock, agricultural, or “fancy” breeding societies (Wood, Orel, 2005; Poczai, Santiago-Blay, 2021; Poczai et al., 2022).

It was reasonable to assume that the most vigorous or prolific organic forms, particularly those with advantageous traits, would be the most abundant. This idea clearly explained the origin of various domesticated varieties and breeds. However, it did not necessarily suggest the emergence of new species, but it was seen as a mechanism for generating new lineages, distinctly differentiated from others, characterized by a homogeneity of traits within the group. This interpretation became increasingly evident during the first half of the 19th century in the works of authors such as John S. Sebright, John Wilkinson, and William Youatt. Their writings provided authoritative support for the concept of selection as the causal process behind the modification of domesticated breeds (Theunissen, 2012, p.183). Thus, the principle of selection, understood as an essential process in the domestication of species, existed before Darwin’s publications.

However, the conceptualization of “artificial selection” and its categorization into “methodical” and “unconscious” selection were original ideas within Darwin’s theoretical analysis, which he began to outline in the 1840s (Alter, 2007, p.65). In works such as the *Pencil sketch* (1842) and the *Essay* (1844), publications that were direct precursors to *On the origin* (Darwin, 1859), Darwin was already addressing domesticated species and the general idea of “selection” (Evans, 1984; Alter, 2007; Theunissen, 2012), elements that would prove to be fundamental components of his subsequent analogy.

For at least fifteen years before the 1859 publication, Darwin had worked continuously on the experimental demonstration of his theory, considering the observation of domesticated forms. With the guidance of William Tegetmeier, a renowned pigeon breeder, he developed various experiments to observe and evaluate the reversion of certain breeds to their ancestral forms, and to demonstrate the inheritance of modified traits, for example, the coloration of wing plumage. He tried similar trials with plant species, though in that context, he analyzed aspects such as variability and the infertility that could arise, whether through hybridization or self-fertilization (Browne, 2008, p.62-63).

Thus, Darwin was widely versed in the techniques, records, and reproductive methods used by breeders and farmers. Consequently, he came to conceive that the cornerstone for the creation of domesticated varieties and breeds was precisely the selection of useful variations and, to a certain extent, directed breeding (Theunissen, 2012, p.182). However, this principle of selection did not link compellingly with the history of species in the wild, at least not until he read Thomas Malthus toward the end of 1838 (Ruse, 1975, p.330-340). We can say that Darwin reflected on domestication and its theoretical implications almost simultaneously with his analysis and integration of Malthusian thought regarding differential survival.

In the first chapter of *On the origin*, Darwin (1859) discussed the nature of variation under domestication, the heritability of such variation, and the distinction between domesticated varieties and wild species. His attention was drawn to the existence of multiple domesticated forms that appeared to be so different from one another, despite belonging to the same taxonomic category (as in the case of the various breeds of pigeons or dogs). According to the experience of various breeders, this diversity was the result of favoring individuals with certain traits and their reproductive isolation. Therefore, the effects of selection (in a context of domestication) were not a speculation or a hypothesis, but a proven fact.

Darwin presented observations about domesticated species as analogous to what occurs in wild populations, specifically, the concept of “selection.” This analogy between artificial and natural selection has frequently been a subject of debate and has received various interpretations (Ruse, 1975; Bartley, 1992; Richards, 1998; Alter, 2007; Gregory, 2009; Hodge, 2012). It is important to note that the analogy proposed by Darwin helped to support the historical reality of common descent and the efficacy of natural selection (Gregory, 2009). However, an interpretation of his arguments reveals certain nuances that are reinforced in his 1868 publication. Our aim here is not to explore this debate in depth, but rather to examine the significance that the concept of “selection”, within the context of domestication, held for Darwin.

Although Darwin used the analogy between the domesticated and the wild as an explanatory resource, it is also true that he employed it as part of a method of scientific investigation in which analogies between phenomena allow them to be grouped if they share the same causal explanation through generalizations. Darwin used the *vera causa* methodology in establishing natural selection as the cause responsible for evolutionary change, drawing upon generalizations from the process of artificial selection as a complete substantiated process (Gildenhuys 2004, p.595; Wilner 2005, p.37; Theunissen 2012, p.181).

In *On the origin*, he stated that selection was the mechanism able to explain the evident divergence between lineages. In particular, “methodical selection” described how the modification of a wild form into a fully domesticated state was achieved, thanks to the differential accumulation of favorable variations under conditions of strict human control and intentionality. Subsequently, he defined the concept of “unconscious selection” to state that this unequal accumulation of traits could occur beyond the scope of human oversight and intervention. Darwin found that, in the history of domestication, unconscious selection had played a fundamental role, as it was inevitable that natural selection would operate even within a human-created environment.

Unconscious selection is a slow, gradual, and non-teleological process that occurs without a specific goal, in contrast to methodical selection (Darwin, 1859, p.34-35). He elaborated this generalization by highlighting that certain variations within populations can be favored even in the absence of human intervention. This can lead to similar results, that is, the formation of races or even more potent effects, such as forming new species. Darwin’s reasoning was an example of inductive generalization, where the selection of heritable variations is sufficient to explain how an ancestral type can evolve into varieties and species (Gildenhuys, 2004, p.598-599).

Alter (2007, p.77) has noted that the analogy between artificial selection and natural selection underwent gradual modification beginning with his early writings (1842 and 1844); it was not until the publication of 1859, and its five subsequent editions, that this concept remained virtually unchanged. A decade later, in the publication of *Variation*, Darwin continued to reinforce this analogy through the copious evidence he presented, and he underscored the role of artificial selection within various models of reproductive management (such as hybridization, crossing, and close interbreeding), while also extrapolating his explanation to natural scenarios to integrate artificial selection as part of a broader spectrum within the evolutionary history of species.

Before Darwin’s work, the principle of artificial selection was understood as a process in which intergenerationally transmitted variation led to phenotypic differentiation, but strictly within the inherent limits of each species, conceived as a fixed entity. It would hardly have been interpreted as a mechanism analogous to that of the formation of new, distinct species independent of the ancestral type. This was initially regarded as a significant weakness in Darwin’s proposal, and not only by his anti-transformist opponents. Alfred R. Wallace, who also proposed the theory of natural selection, deemed the evidence derived from domestication to be inadmissible as support for arguments regarding the generation of new species (Richards, 1998, p.106; Bock, 2009, p.8; Varno, 2011, p.23).

In *Variation*, Darwin (1868) emphasized the nearly simultaneous emergence of methodical selection and unconscious selection. Unconscious selection is the process by which specific traits of interest are preserved in individuals, but others (not necessarily useful) are also indirectly preserved. As these traits would affect through generations, they can influence the differentiation of races, even though this was not the purpose of the breeder (Darwin, 1868, p.212). For this reason, unconscious selection may be even more significant than methodical selection. Without a defined objective, its potential for modification was likely greater than that of methodical selection. By not focusing on a specific end goal, this concept could bridge the gap between methodical human intervention (artificial selection) and nature immersed in chance (natural selection) (Alter, 2007, p.60). This interpretation allows us to understand natural selection and artificial selection, both methodical and unconscious, as part of a continuous model rather than a categorical dichotomy (Gregory, 2009, p.6).

According to Darwin, artificial selection generated divergence that originated in modern breeds and varieties, departing from their wild ancestors (Darwin, 1868, p.239). Even though in the domestication process, this separation of lineages could not be so intense, it only implied the change in a subset of individuals from an original species, and the main cause was in different selective contexts. For wild forms, these pressures are related to survival and reproduction in nature, whereas for cultivated forms, the pressures are imposed by domesticators (Gregory, 2009, p.21). Based on this reasoning, Darwin concluded that this divergence from the ancestral type, across a deeper timescale and within more complex contexts, could explain the origin of all species.

## Methods of selective breeding and their application to the artificial selection analogy-natural selection

Although Darwin always highlighted the principle of selection as the cause of the improvement of breeds (Theunissen, 2012; Del Savio, Mameli, 2020, p.21), he was also interested in explaining the role of reproductive methods in the development of domesticated forms (Derry, 2015, p.20; Bradshaw, 2016, p.35). Practices such as hybridization, crossing, and close interbreeding, which involved not only the management of available resources (food, soil type, fertilizers, space, fencing, among others) but also the selection of the “best” organisms, specifically the most striking or exotic ones, were used to facilitate their reproduction and, consequently, to replicate those characteristics in their offspring.

The interpretation that Darwin (1868) developed regarding these techniques in the second volume of *Variation* will be discussed below. By integrating these concepts into his analogy, he attempted to delineate scenarios applicable to both domesticated and wild forms.

### Hybridization

Hybridization, as a strategy in the management of domesticated forms, was based on reproduction between individuals of different species. However, the term “hybrid” eventually refers to an organism resulting from a cross between individuals of the same breed or variety, or between members of different breeds that were considered to belong to the same species (Wood, 2003, p.38).

In both *On the origin* and *Variation*, Darwin used the word *mongrel* to refer specifically to offspring obtained from crossing different varieties considered to be the same species, and the concept of *hybrid* for crossing different species (Theunissen, 2012, p.185). However, this categorization, though useful in practical terms, was indirectly challenged by Darwin, who argued that the criteria (morphological similarity, interfertility, and sterility) distinguishing varieties from species are not exact parameters, but rather vary along a spectrum linked to the life history of individuals and the evolutionary history of the species.

The mechanisms behind the intermediate or blended inheritance of traits in hybrid individuals were not precisely understood. However, based on the concept of fluid inheritance, it was generally accepted that hybridization resulted in sterile plants with intermediate phenotypes (Müller-Wille, 2003, p.52). Nevertheless, numerous empirical and experimental lines of evidence provided by breeders and naturalists showed that the biological phenomenon of hybridization was far more complex than simply producing sterile offspring and intermediate morphologies.

In the first edition of *On the origin*, Darwin (1859) dedicated a chapter titled “Hybridism” to discussing the criteria most frequently invoked to taxonomically establish and identify the category of species, and which, ultimately, were used to defend the immutability of living beings: reproductive compatibility, as evidenced by interfertility and the sterility of hybrid crosses. According to Darwin, the sterility in the products of hybrid crosses could not be explained as a result of natural selection, since it would not be advantageous for organisms to lose the capacity to leave offspring (p.245). However, the outcome of hybridization did not always result in completely sterile offspring. He suggested that when sterility was observed in hybrid organisms, it was possible due to the self-fertilization promoted over several generations to replicate that lineage (p.248).

In *Variation*, Darwin (1868) reviewed the investigations by both naturalists and plant breeders since the late eighteenth century. Most of his discussions regarding hybridization remained entirely unchanged. However, this publication introduced a new perspective on variability and its potential causes. For example, he noted that, for some experts, hybridization represented a source of induced variation (like the crossing of varieties) (p.180), while emphasizing that it did not constitute the ultimate cause of this process.

According to Darwin (1868), the patterns observed in crossing, whether intra- or intervarietal, were the same as those to be expected in hybridization (except for the phenomenon of sterility-fertility). Therefore, sexual reproduction in general, whether through crossing or hybridization, represented a source of variation in the resulting mongrel or hybrid offspring, respectively. This proposition was directly linked to the recognized fact that natural varieties, species, and domesticated varieties differ only in degree, not in type (Varno, 2011, p.26).

In comparing crosses between varieties and species, Darwin aimed to show that the processes observed in both categories were identical and led to similar expected patterns. He further stated that these same general processes applied to mongrels and hybrids as they did to domesticated forms and wild species. This demonstrated that species are not static entities. Therefore, he maintained that strict criteria could not be applied to identify them. Whether in a natural state or under domestication, organisms inherently vary and exist within distinct, dynamic, and selective contexts, factors that drive the differentiation and specialization of populations.

Hybridization was a model that enabled Darwin to challenge traditional reasoning regarding the theoretical and methodological distinction between species and varieties. Thanks to advances in experimentation, he showed that the reproductive barriers between species and varieties were caused by a law of reproduction common to both categories. Consequently, neither sterility nor fertility offered a consistent criterion for distinguishing between species and varieties (Wilkins, 2009, p.151; Varno, 2011, p.27). This explains why he viewed varieties as “incipient species” (Darwin, 1859, p.52, 1868, p.185-186), since they ultimately become genuine species.

### Crossing

Crossing is a technique used by domesticators in the sexual reproduction of organisms, in which mating between individuals of the same breed or variety is controlled and directed (Wood, 2003, p.30). If it could be established that two morphologically similar individuals were merely varieties of the same species, then it was predictable that reproduction would be entirely possible, without any barriers of any kind.

During the era of technological advancements, various breeders’ associations began collaborating closely with naturalists, anatomists, and physiologists to develop explanations regarding variation, development, trait transmission, and breed modification. Together, they described the general processes underlying these phenomena. Among the earliest of these organizations was the Dishley Society, founded in 1783 by Robert Bakewell along with livestock breeders from central England, aimed at improving sheep breeds (Wood, 2003, p.21). Another group was the Society for the Improvement of British Wool, established in Edinburgh in 1791 (Poczai et al., 2022, p.6). These pioneering associations, alongside industrialization and the context of territorial conflict, led the emergence of numerous similar institutions across continental Europe. Their shared interest in understanding and managing the processes of heredity fostered a network of material culture among local populations, involving exchanges with foreign entities that went beyond the mere commercialization (Wood, 2003; Wood, Orel, 2005; Poczai, Santiago-Blay, 2022; Poczai et al., 2022). Many explanations derived from observations and experiments with domesticated species were applied to natural species, those existing beyond human control, to address their origins and to explore how significant modifications might occur through crossing (Poczai, Santiago-Blay, 2022, p.7).

Although domesticators and naturalists were aware of the emergence of spontaneous variations (morphologically recognizable) in the constitution of individuals (Wood, 2003, p.35), the most evident source of variability was intervarietal crossing. The great diversity observed, at least in domestic stocks, was attributed to the proximity in which varieties or breeds were cultivated or raised, as this facilitated crossing (which was not always intentionally directed) (Oghina-Pavie, 2015, p.56). Crossing was regarded by Darwin as a source of variability (Browne, 1980, p.66), as was hybridization (Winter, 2000, p.431).

Darwin observed that crossing leads to two opposing patterns. On the one hand, when crosses are made between closely related individuals, it can create homogeneity and prevent the formation of new races. On the other hand, it could also lead to the modification of old races to generate new variants when crossing was conducted between different races (Theunissen, 2012, p.195). This second tendency introduces the variation needed for trait differentiation and an increase in the constitutional vigor and fertility of the hybrid offspring. These factors, according to Darwin, would contribute to the hybrids multiplying more rapidly than the pure parental races (Darwin, 1868, p.85-86).

Crossing can introduce variation in domesticated organisms, as mixing two purebred lines often leads to intermediate phenotypes. However, more variable traits can emerge in subsequent generations (Wood, 2003, p.38). This variation is beneficial because new traits can enable more systematic selection, ultimately resulting in the development of new breeds (Wood, 2003, p.39; Poczai, Santiago-Blay, 2022, p.6). Darwin applied this concept within his broader theory, not only to domesticated species but also to wild ones. He argued that free crossing among individuals could produce heritable variations, potentially offering advantages in natural environments (Darwin, 1868, p.90).

In other words, Darwin defended the generalization of the principle of selection, applying his argument within a model in which free crossing, under wild conditions, also played a role in the history of species, providing heritable and advantageous variation that contributed to the divergence of lineages. According to the experience of breeders, it was common for one of the parental forms involved in a cross to possess a greater capacity to transmit its traits to the offspring, that is, it exhibited “prepotency.” Indigenous breeds were better adapted to local conditions compared to introduced forms (Wood, 2003). Darwin (1868, p.89) demonstrated that natural selection was not suppressed, even under conditions of domestication.

### Close interbreeding

The crossing of closely related individuals, not merely belonging to the same variety, but sharing a close blood tie, became a common practice in the management of domesticated organisms starting in the late 18th century, and intensified throughout the 19th century (Orel, Wood, 1981; Wood, 2003; Derry, 2015; Poczai, Santiago-Blay, 2021). In animals, the technique involved selecting organisms that exhibited traits of interest and, much like in a standard cross, directing their mating to produce offspring. However, the chosen individuals were required to share a very close genealogical relationship (father and daughter, mother and son, brother and sister etc.). In plants, the technique was fundamentally the same. The most closely related specimens could be crossed, although in this type of organism, this proximity could extend to the degree of self-fertilization.

As previously mentioned, crossing introduced variability while also enabling the faithful replication of typical traits within a breed through the reproduction of closely related organisms, specifically, interbreeding. This process allowed for the preservation of characteristics and limited variation. The method known as “breeding in-and-in” was developed by the sheep-breeding expert Robert Bakewell, who was a pioneer in the implementation of this system, as part of a mass-production model that was applied to the improvement of various livestock. This strategy combined the careful selection of vigorous, fertile individuals with notable traits, followed by reproduction among blood relatives (Orel, Wood, 1981, p.153-154).

This technique became indispensable in intensive breeding because, through this procedure, homogeneous or pure breeds were obtained (Wood, 2003, p.32; Stoykovich, 2010, p.36; Poczai, Santiago-Blay, 2021, p.5). In the history of those well-characterized breeds, whose economic importance was high, a stage of close interbreeding was necessary to standardize the phenotype associated with that aesthetic and/or economic value.

Since *On the origin*, Darwin had noted that close interbreeding resulted in a decline in the constitutional vigor of the offspring, as well as sterility. However, this was not a subject he explored in depth beyond citing general observations from experts in animal breeding. In *Variation*, he expanded upon the discussion of these practices, optimized by the domesticators of the era. He pointed out that interbreeding allowed for the observation of various biological phenomena in domesticated organisms. These phenomena could readily be extrapolated to natural populations, suggesting that this process likely played a significant role in the history of species in general, not merely of domesticated ones.

Under wild conditions, Darwin (1868, p.114-115) explained that the persistence of interbreeding within a population was linked to factors such as low population density, a low probability of hybridization, and a restricted spatial distribution. He compared wild British cattle (confined to a private estate) and semi-wild cattle in Paraguay (subject to less control regarding free crossing). Through this comparison, he sought to demonstrate that fertility is diminished in populations where close interbreeding has occurred over generations, at least as the available records allowed us to determine (p.119).

In domesticated organisms, interbreeding was the means to get purebred improvement. However, this practice was generally associated with a loss of fertility, malformations, or impaired capabilities (Wood, 2003, p.31; Poczai, Santiago-Blay, 2021, p.6-7). This was the case with Shorthorn cattle, native to Northeast England, which had been extensively interbred from the late 18th century through the mid-19th century, and which produced a higher number of offspring with malformations compared to other breeds (Darwin, 1868, p.118).

However, the time to manifest adverse effects could sometimes be prolonged, as the accumulation of detrimental traits was gradual. Darwin (1868, p.115-116) noted that during the first half of the nineteenth century, this practice had intensified, and that a precautionary measure adopted by some experts consisted of moderating the use of interbreeding among close relatives while favoring occasional crosses with individuals of the same breed, though not closely related genealogically, as this was a proven technique for imparting vigor to the offspring.

Once the desired morphology had been successfully “sculpted,” interbreeding allowed for the attainment of a certain degree of hereditary stability by preventing, to the greatest extent possible, the emergence of variations that deviated from that specific typology. However, breeders were acutely aware of the constant appearance of fortuitous characteristics, as well as the adverse effects inherent to interbreeding. They understood that, to a certain extent, these unwanted consequences lay beyond the reach of selective control, for even if an individual did not outwardly display a defect, one could not simply assume that the trait was not carried internally, nor rule out the possibility that such a disadvantage might be inherited (Wood, Orel, 2005, p.245).

The cause of the degeneration and infertility resulting from consanguinity was not precisely understood until the development of Mendelian inheritance as a field of research in the 20th century. However, it was hypothesized that, in interbreeding, closely related individuals who reproduced were more likely, by virtue of their ancestry, to possess the same deleterious variations, which would then be intensified in their offspring. In the crossing of different races, the parents possessed distinct variations that could be mutually compensated in their progeny (Zirkle, 1952, p.11).

## The meaning of domestication in *Variation*

By the second half of the 18th century, new concepts were developed, spurred by a growing confidence in human intervention in nature, an outlook that entailed interpreting the process of domestication as the human domination of the natural world. The breeding and cultivation practices developed and refined between the 18th and 19th centuries reinforced these notions, leading to a view of domestication as the management and systematization of organism handling for improvement, guided by specific criteria and standards. By the second half of the 19th century, the term “domestication” began to circulate within scientific and economic parlance in specialized settings, such as various acclimatization societies, where the defining criterion was reproduction under human control (Bogaard et al., 2021, p.5), a parameter that has persisted in numerous modern definitions.

Although domesticated forms have been examined within the framework of the transformist debate since the early nineteenth century, there has not been an explicit conceptualization of the term “domestication” in this new context. The similarities between domesticated animals and their wild counterparts are evident, often suggesting a common origin within the same species. However, questions remain regarding how they diverged, when this divergence occurred, where it took place, and whether the potential for change is unique to domesticated forms or a characteristic of all species. These inquiries have typically been addressed in isolation.

For Darwin, the concept of “domestication” meant an experimental process of transformation in which organisms adapt to a human-fabricated environment. The transition from the wild to the domesticated, when viewed on a geological timescale, might not reveal a change pronounced enough to warrant asserting the emergence of a new species. However, on a temporal scale limited to a few generations, the modifications would indeed be significant. This applies not only when considering changes in animal behavior or evident alterations in the organisms’ physical appearance. Domestication would be deemed achieved once certain attributes, such as fecundity, variability, and plasticity, were attained effortlessly or even enhanced. This would demonstrate an adjustment to the modified conditions of life (Darwin, 1868, p.405). Such transformations, when contrasted with the typology of wild forms, evidenced a historical divergence of lineages, a divergence replicated generation after generation, which reinforced a reproductive separation and demonstrated that this process could proceed in numerous directions, depending on varying circumstances.

Asa Gray, one of Darwin’s most important correspondents in the United States, emphasized the importance of Darwin’s idea that varieties can be seen as incipient species. He closely examined domesticated organisms and what they reveal about the origins of different forms. The ability to transform these organisms into a kind of “monstrosity” (even if such forms could neither originate nor persist in nature) clearly demonstrated the limitless power of variation and highlighted its universal tendency. This tendency exists not only in domesticated organisms but also in wild ones, although each is influenced by specific characteristics linked to their environment and particular history (Gray, 1876, p.26).

The concept of “artificial selection,” which Darwin used to support his theory, referred to a process of trait accumulation brought about by human intervention (whether intentional or not) in the reproduction of organisms, aimed at generating character divergence. The attributes in question typically involved docility in the case of animals, fecundity, or the persistence of juvenile traits into adulthood; in plants, they included rapid growth and a short life cycle, tolerance to varying climatic conditions, and the production of large or easily harvestable seeds, among others. The isolation and replication of these characteristics, that is, through the selection of which organisms would be permitted to reproduce, would result in morphological differentiation and specialization. Fundamentally, this process could occur in both natural selection and any category of artificial selection.

For Darwin, the fundamental factor behind domestication and natural transformation was the general principle of selection. In the first chapter of *On the origin*, which he expanded upon in *Variation*, he argued that domestication demonstrated the occurrence of changes (phenotypic, hereditary, and ecological) within subpopulations undergoing processes linked to human management. By generalizing these mechanisms, he was able to establish the similarities between domestication and the evolutionary process, as in both cases, the most powerful causal element was the principle of selection (Gildenhuys, 2004, p.596). This theoretical framework, developed by Darwin, allowed domestication and artificial selection to be understood as highly specific evolutionary processes, giving them a new meaning.

To this end, he describes domestication as a historical process involving distinct stages, each characterized by different key mechanisms. He connected various components (variation, heredity, and reproduction) that were necessary to explain both unconscious and methodical selection. This process is carried out through strategies of selective breeding (hybridization, crossing, and close interbreeding), which help delineate these potential stages in the history of a domesticated species. Domestication must begin with a group of wild individuals, whether they belong to the same species or are the result of prior hybridization (a fact that would have supported the notion that hybridization occurs under wild conditions before human intervention). During this initial stage, either interspecific crossing (hybridization) or intraspecific crossing introduces variation and enhances vigor, both of which are valuable to humans. From these hybrid or crossbred specimens, the most desirable phenotypes could be selected, and distinct breeds could be generated through intervarietal crosses. The final stage involves the homogenization of a breed, achieved by limiting variation, specifically by employing inbreeding over multiple generations, and consistently selecting only the best specimens.

These processes were documented in ancient practices of breeding and cultivation, yet abundant records regarding their occurrence in wild conditions did not exist. Nevertheless, Darwin investigated and considered several examples of wild or semi-wild populations in which there was evidence of hybridization, crossing, or close interbreeding. He identified the circumstances under which such events might occur, whether through geographical isolation resulting from geological alterations, migration, or the colonization of new territories, while consistently emphasizing the principle of selection as the fundamental cause of differentiation. In other words, whether in the history of a domesticated species or in that of a species transformed in the wild, the fundamental processes driving their modification were the same.

Darwin’s approach was innovative because it combined various elements that often seemed unconnected, such as variation, reproduction with heredity, and adaptation to the environment, into a unifying explanation: wild organisms undergo processes of natural selection, while domesticated organisms may experience both natural and artificial selection, even simultaneously. This generates observable and measurable changes in organisms, leading to the differentiation of populations (races and species) (Darwin, 1868, p.409-410).

Darwin’s reinterpretation of domestication and artificial selection, as well as how he integrated these ideas into his overall framework, had significant implications for the various interpretations of his theory. Both his opponents and supporters saw the analogy between domestication and evolution as contradictory and evidence that conflicted with the conclusions he aimed to support (Richards, 1998; Gregory, 2009, p.7). However, the evidence gathered from domesticated models bolstered Darwinian interpretations. As a result, domestication helped validate his vision and persuaded some of his contemporaries, such as Joseph D. Hooker (Bellon, 2006, p.31) and Alphonse de Candolle (Alvarez-Tostado et al., 2024, p.463).

Darwin always advocated the relevance of his hypothesis. However, other underlying mechanisms, similar to artificial selection in their capacity to drive (non-adaptive) change, escaped his analysis. He suggested an initial scenario of domestication in which humans began to isolate managed resources from free-living populations, particularly when these resources were transported beyond the natural geographic range of their wild progenitors. Today, we understand that other processes also contribute to domestication. In addition to artificial selection, mechanisms such as genetic drift and gene flow come into play, not only at the onset of domestication but recurrently throughout the history of a domesticated species (Gregory, 2009; Allaby, Ware, Kistler, 2018; Moreno-Letelier et al., 2020). In fact, modern approaches suggest that artificial selection, specifically through deliberate breeding to foster particular traits, represents a relatively late development in the history of most domesticated species (Zeder, 2012; Zeller, Göttert, 2019).

## Final considerations

Following the publication of *On the origin*, Darwin dedicated himself to defending the scientific importance of the principle of selection. In his work *Variation*, he aimed to strengthen the causal efficacy of natural selection by highlighting, within his conceptual framework, the proven causality of artificial selection regarding the tendency toward differentiation among races, as observed in contexts of animal breeding and plant cultivation. By demonstrating how selective breeding leads to the divergence of traits among subpopulations, he argued that natural selection is a more powerful force, possessing more definitive scope, such as the transformation of species.

Implicit in Darwin’s perspective on domestication was human control and the management of species as exploitable resources, not merely by conceiving of them as livestock, crops, or material and even aesthetic sources. Biological processes represented a form of resource: the natural capacity to vary, the ability to inherit traits, the capacity to reproduce morphological types, the ability to adjust to new environments, or the capacity to be modified. These mechanisms, which for a long time remained mysterious and difficult to manipulate, constituted the raw material enabling domestication to occur. Considering the historical universality of these components likely facilitated Darwin’s conception of domestication as a particular type of evolutionary scenario, temporally bounded, in which organisms adapt to a human-fabricated environment.

Before the consolidation of evolutionary thought, the management of domesticated resources involved intervening in and manipulating the reproduction of individual organisms. The methods used for these purposes included hybridization, crossing, and close interbreeding. Each of these techniques facilitated the efficient management of resources, depending on the specific objective being pursued. Hybridization allowed for the introduction of interspecific variation and the blending of traits from distinct lineages; indeed, it was even hypothesized that entirely new species could be created through this process. Crossing also introduced variation, albeit within narrower limits, almost invariably producing inter-fertile individuals, a characteristic that enabled iterative replication for the production of specific breeds or varieties. Close interbreeding stood at the opposite end of the spectrum from hybridization, fostering homogeneity among domesticated individuals and thereby virtually guaranteeing the consistent production of traits of interest.

The idea of domestication, the general concept of selection, and strategies for reproductive management already held significant importance within the body of knowledge possessed by domesticators and naturalists, even before the emergence of evolutionary theory. However, these concepts underwent a complete reinterpretation when viewed through the lens of Darwinian theory. Today, it is accepted that both domestication (modification and adaptation to human-mediated environments) and artificial selection (the accumulation of specific variations through reproductive control) constitute evolutionary processes in their own right.

## Data Availability

They are not in the repository.
