# Supplementary material for: The concepts of “domestication” and “artificial selection” and their significance in the work of Charles Darwin
Source: Hist Cienc Saude Manguinhos. 2026 Aug 14;33:e2026033. doi: 10.1590/S0104-59702026000100033en (PMC13475949; doi:10.1590/S0104-59702026000100033en)
Supplement: Translation

# Translation: Los conceptos “domesticación” y “selección artificial” y su significado en la obra de Charles Darwin

**DOI:** 10.1590/S0104-59702026000100033

**Authors:** Miriam Rebeca Alvarez Tostado Reyes, Fabiola Juárez-Barrera, Alfredo Bueno-Hernández

**Affiliations:** i Coordinación de Estudios de Posgrado, Programa de Posgrado en Ciencias Biológicas, Universidad Nacional Autónoma de México. Ciudad de México – México. mirirbk29@gmail.com; ii Profesora, Facultad de Estudios Superiores Zaragoza, Universidad Nacional Autónoma de México. Ciudad de México – México. fabiolajuarezunam@gmail.com; iii In memoriam

**Keywords:** Domesticación, Selección artificial, Hibridación, Cruzamiento, Entrecruzamiento

## Abstract

Este análisis revisa los términos “domesticación” y “selección”, contenidos en *The variation of animals and plants under domestication*, de Darwin, y el significado que éste otorgó a estas nociones. Este enfoque se centra en cómo Darwin se basó en los conocimientos generados siglos antes por expertos en cría y cultivo, para modificar las definiciones prevalecientes y utilizar este cambio semántico como los instrumentos más importantes para defender su teoría. La construcción de la “analogía” entre selección natural y artificial no fue un mero recurso heurístico, sino el resultado de una generalización inductiva, sostenida en evidencia incontestable. Ambos conceptos se reinterpretaron, a la luz del transmutacionismo darwiniano, como escenarios evolutivos específicos.

La connotación evolutiva de “domesticación” se remonta al siglo XIX, periodo en el que surgió y se desarrolló el debate entre la doctrina fijista y la visión transmutacionista de las especies. Fue particularmente la obra darwiniana en donde se originó este nuevo significado, aunque antes de Darwin, otros naturalistas ya habían considerado este proceso como evidencia de la mutabilidad de las especies (Burkhardt, 1995; Corsi, 2005; Galera, 2016; Friedman, Endress, 2020). En contraste, hubo autores que percibían a las formas domesticadas como producciones artificiales cuyas modificaciones eran reversibles y, por lo tanto, no trascendían el carácter esencialmente fijo de la categoría de especie (Corsi, 2004 **,** p.224, 2012, p.31).

Para Darwin, la domesticación no solo representó un modelo de la tendencia a la modificación gradual, sino que en sí misma se trataba de un proceso transformativo. Lo que se investiga en este trabajo son dos cuestiones principales: ¿cuál fue el significado que Darwin le otorgó a las nociones de “domesticación” y “selección artificial”? y ¿cuál fue la importancia de estos conceptos en su teoría?

Examinar la resignificación planteada por Darwin es importante desde diferentes perspectivas. En primer lugar, nuestro análisis histórico nos permite apreciar que, en contraste con Coppinger y Smith (1983), Darwin en realidad no consideró el proceso de domesticación como algo separado de la naturaleza o como una simple analogía metafórica de la evolución de las especies (Richards, 1998; Ritvo, 2013). En segundo lugar, nuestra aportación es pertinente al poner en contexto el uso que dio al concepto de “domesticación” en algunas de sus producciones científicas más relevantes, ya que representa el origen del componente evolutivo de este término. Aunque el papel de la domesticación y la selección artificial en el pensamiento darwiniano se ha explorado ampliamente (Ruse, 1975; Evans, 1984; Bartley, 1992; Browne, 2008; Varno, 2011; Hodge, 2012; Theunissen, 2012), nuestro enfoque se centra en cómo Darwin se basó en los hechos probados por la experiencia de la cría y el cultivo para modificar el significado de estos conceptos preexistentes. Este cambio semántico se convirtió en uno de los instrumentos más importantes para la defensa de su teoría. Ese nuevo sentido que otorgó a “domesticación” y “selección artificial” contribuyó a ver a los diferentes mecanismos involucrados en la formación de las razas domesticadas como procesos universales que también actúan en las especies en estado silvestre.

El análisis histórico de estos componentes contenidos en su obra permitirá tener una comprensión más amplia y matizada de cómo es que identificó un significado innovador, así como entender la importancia de estos componentes en su teoría, ya que demostraban con evidencia suficiente que las variedades tienden a alejarse de su tipo original, en función de su ambiente y que la variación, como un atributo universal, representaba una tendencia potencialmente ilimitada.

Previo al evolucionismo, se reconocía la existencia de condiciones selectivas sobre el recurso gestionado, que se asemejaban a las presiones que experimentan los organismos en estado silvestre, aunque con diferencias en la escala temporal. En diferentes regiones de Europa, desde finales del siglo XVIII se había producido un desarrollo significativo en la teoría de la “selección” (aun sin la llegada del pensamiento darwiniano), originado desde las prácticas de agricultores y criadores (Theunissen, 2012; Hodge, 2012, Derry, 2015; Poczai, Santiago-Blay, 2021). Un importante ejemplo de ello se presentó en los territorios de Europa central, particularmente en Moravia (actual Republica Checa), donde la organización de criadores de ovejas dio origen a sociedades privadas en las que se discutía el mejoramiento en las técnicas reproductivas del ganado. En estos espacios surgió el concepto *künstliche Zuchtwahl*, cuya traducción literal es “selección artificial”, propuesto por Christian Carl André en 1812, para referirse a la modificación de forma y características en los animales y que, en conjunto con técnicas de reproducción, habría generado las razas domésticas de las que se tenía conocimiento (Poczai, Santiago-Blay, 2022, p.11). Las explicaciones surgidas de registros, intercambios y experimentación en el mejoramiento de razas y variedades frecuentemente se extendieron hacia los debates y teorías científicas, debido a que los efectos que la selección provocaba en los organismos bajo domesticación proporcionaban importante información sobre los patrones de variación y herencia.

Aunque Charles Darwin no fue el único naturalista que prestó atención a las especies domesticadas para sostener explicaciones sobre la mutabilidad de las especies, es posible decir que fue uno de los primeros autores en considerar el proceso de domesticación como una práctica experimental que permitía demostrar la modificación y sucesión de las especies como resultado de la concatenación de ciertos componentes: variabilidad, heredabilidad y supervivencia-reproducción diferencial.

La premisa de la que se parte es que la revisión de publicaciones y textos dedicados a las prácticas y teorías de la domesticación y su significado, durante el siglo XIX, nos proporcionará una perspectiva histórica sobre esta disciplina, que permitirá a su vez analizar su relación con el evolucionismo a medida que se integró al nuevo paradigma. En ese sentido, la obra *The variation of animals and plants under domestication*, de Charles Darwin (1868), representa una fuente útil para este propósito por dos razones: (1) antes de su publicación, el debate sobre la evolución de las especies y los mecanismos subyacentes ya se encontraba en la atmósfera intelectual europea, ya que desde de 1809, con la publicación de *Philosophie zoologique*, de Jean-Baptiste Lamarck (1809), y durante las cinco décadas siguientes, se habían publicado diversas obras destinadas a discutir la idea general de la transformación de las especies (Corsi, 2005, p.68; Galera, 2017, p.29); (2) en *Variation*, Darwin (1868) presentó numerosas evidencias experimentales y empíricas proporcionadas por diversos autores, algunos dedicados a la cría de ganado o a la agricultura, así como de diversos naturalistas interesados en los mecanismos de variación, herencia, reproducción y/o modificación. Por ello, su investigación representa un muestreo de las técnicas especializadas y el desarrollo teórico en torno a la domesticación durante este periodo de transición. Con la interpretación darwiniana, el estudio científico de la domesticación hizo posible concebir este proceso como un modelo que permitía observar y comprender los cambios en los seres vivos, pues sostuvo que aquellos componentes necesarios en la domesticación son esencialmente los mismos elementos que se necesitan para que ocurra la mutabilidad de las especies (Varno, 2011, p.42).

Ya que la domesticación implica la selección de rasgos en los individuos, es común asumir que “domesticación” y “selección artificial” pueden ser términos equivalentes. *Variation* fue una de las primeras publicaciones en abordar la relación entre estos procesos e interpretarlos como modelos evolutivos. Aunque sin duda son nociones estrechamente relacionadas, nuestra exploración nos permite observar que, si bien Darwin no enunció directamente una distinción entre ambas nociones, siempre aplicó un uso reconociblemente diferente para cada término.

Comenzaremos a revisar la importancia de la idea general de selección surgida de los expertos en cría y cultivo, y cómo Darwin interpretó este concepto al aplicarlo a su noción de selección artificial. También se analizará el papel de las prácticas de reproducción selectiva, tales como la hibridación, el cruzamiento y el entrecruzamiento, como el conjunto de evidencias más sólidas con las que Darwin explicó el papel fundamental de la variación para la diferenciación morfológica entre variedades. Las generalizaciones extraídas de estos hechos le permitieron extender su argumento hacia escenarios silvestres. Finalmente, discutiremos la interpretación que Darwin hizo del concepto “domesticación”, como un proceso evolutivo particular, demostrando que ciertas creencias – como pensar que las variaciones siempre tenían un límite, o que eran reversibles, y que las variedades nunca traspasaban el límite de especie – eran concepciones que se oponían a los hechos. La mutabilidad de los seres vivos se presentaba en todo tipo de escenarios, incluso si el contexto estaba restringido a condiciones específicas como la participación humana y la escala temporal.

## El principio de “selección” en el pensamiento darwiniano

Desde una etapa temprana, poco después de su regreso del viaje en el HSM Beagle, la domesticación tuvo un lugar importante en las reflexiones transformistas de Darwin (Ruse, 1975; Evans, 1984; Bartley, 1992; Varno, 2011; Theunissen, 2012). Comenzó a examinar cuidadosamente este indicio para la construcción de su teoría en sus cuadernos de notas, particularmente en el tomo C (febrero-julio de 1838) (Ruse, 1975, p.344; Evans, 1984, p.122). Asimismo, en 1840, tras la lectura de *Cattle, their breeds, management and disease*, de William Youatt (1834), Darwin comenzó a emplear los términos *picking* y *selection* para referirse a la naturaleza de las variedades y su relación con el mejoramiento de plantas y animales (Evans, 1984).

La idea general de “selección” estaba contenida en la literatura especializada sobre reproducción selectiva, desde el siglo XVIII (Wood, 2003, p.27; Poczai, Santiago-Blay, 2021, p.5, 2022, p.6). Esta noción puntualizaba la necesidad de escoger/cosechar los individuos más notables, más productivos o peculiares, pues se esperaba un resultado casi invariable, atribuido a la antigua idea de “lo semejante engendra lo semejante” (Wilkinson, 1820, p.20; Youatt, 1834, p.60). Hacia el siglo XIX siguió una mayor expansión de las perspectivas hacia la teoría de la selección y sus múltiples aplicaciones. Se establecieron estructuras administrativas para el cultivo y la cría, ya fuese mediante intervenciones gubernamentales para el desarrollo de los sectores agrarios (Macdonald, 2017, p.21), o bien, mediante la organización de particulares en sociedades ganaderas, agrícolas o para la cría de fantasía (Wood, Orel, 2005; Poczai, Santiago-Blay, 2021; Poczai et al., 2022).

Era lógico asumir que las formas orgánicas más vigorosas, más fecundas, o que poseyeran rasgos favorecedores, fuesen las más abundantes. Esta idea explicaba con claridad el origen de las diferentes variedades y razas domesticadas, y no implicaba necesariamente el origen de nuevas especies, sino que se interpretaba como un mecanismo para originar nuevas estirpes bien diferenciadas de otras, con homogeneidad en los rasgos dentro de la agrupación. Esa interpretación se volvió más evidente en la primera mitad del siglo XIX en autores como John S. Sebright, John Wilkinson y William Youatt, cuyas obras fueron respaldos de autoridad en torno a la idea de selección como proceso causal de la modificación de razas domesticadas (Theunissen, 2012, p.183). Por lo tanto, el principio de selección, como un proceso esencial en la domesticación de las especies, antecedió a las publicaciones de Darwin.

Sin embargo, la conceptualización de la “selección artificial” y su categorización en selección “metódica” e “inconsciente” fueron ideas originales en el análisis teórico de Darwin, las cuales comenzó a delinear en la década de 1840 (Alter, 2007, p.65). En obras como *Pencil sketch* (de 1842) y el *Essay* (de 1844), publicaciones que fueron un antecedente directo de *On the origin* (Darwin, 1859), Darwin ya abordaba las especies domesticadas y la noción general de “selección” (Evans, 1984; Alter, 2007; Theunissen, 2012), los cuales fueron componentes fundamentales para su analogía posterior.

Desde al menos 15 años antes de la publicación de 1859, Darwin había trabajado constantemente en la demostración experimental de su teoría, tomando en cuenta la observación de formas domesticadas. Con asesoría de William Tegetmeier, un reconocido criador de palomas, desarrolló diversos experimentos para observar y evaluar la regresión de algunas razas a su forma antepasada, así como demostrar la herencia de los caracteres modificados, por ejemplo, la coloración en el plumaje de las alas. Con especies vegetales intentó ensayos parecidos, pero allí pretendía analizar aspectos como la variabilidad y la infertilidad que llegaba a presentarse en los organismos vegetales, ya fuese por hibridación o por autofertilización (Browne, 2008, p.62-63).

Es decir, Darwin estaba ampliamente versado en técnicas, registros y métodos reproductivos empleados por criadores y agricultores, por lo que llegó a concebir que la piedra angular para la creación de variedades y razas domesticadas era precisamente la selección de variaciones útiles y, en cierta medida, la reproducción dirigida (Theunissen, 2012, p.182). Sin embargo, por sí solo, este principio de selección no se vinculaba contundentemente con la historia de las especies en condiciones silvestres, al menos no hasta que leyó a Thomas Malthus hacia finales de 1838 (Ruse, 1975, p.330-340). Es posible decir que Darwin reflexionó sobre la domesticación y sus implicaciones teóricas casi al mismo tiempo que analizaba e integraba el pensamiento malthusiano sobre la supervivencia diferencial.

En el primer capítulo de *On the origin*, Darwin (1859) inició discutiendo la naturaleza de la variación bajo domesticación, la heredabilidad de tal variación y la distinción entre variedades domesticadas y especies silvestres. Además, llamó su atención la existencia de las múltiples formas domesticadas que parecían ser tan diferentes entre sí, a pesar de pertenecer a la misma categoría taxonómica (como en el caso de las diversas razas de palomas o de perros). De acuerdo con la experiencia de los diferentes criadores, esta diversidad era el resultado del favorecimiento de individuos con ciertos rasgos y su aislamiento reproductivo. Por lo tanto, los efectos de la selección (en un contexto de domesticación) no eran una especulación o una hipótesis, sino un hecho comprobado.

Darwin presentó las observaciones sobre las especies domesticadas como hechos análogos a lo que ocurre en las poblaciones silvestres, especialmente la ocurrencia de “selección”. Esta apreciación sobre la analogía entre la selección artificial y la natural ha sido frecuentemente un motivo de debate y ha estado sujeta a diferentes interpretaciones (Ruse, 1975; Bartley, 1992; Richards, 1998; Alter, 2007; Gregory, 2009; Hodge, 2012). Es posible notar que la analogía planteada por Darwin sirvió para defender la realidad histórica de la descendencia común y la eficacia de la selección natural (Gregory, 2009). Sin embargo, la lectura que podemos hacer de sus argumentos tiene ciertos matices que parecen reforzarse en la publicación de 1868. Nuestra intención no es profundizar en este debate, sino examinar el significado que pudo tener para Darwin el concepto de “selección” dentro del marco de la domesticación.

Aunque Darwin utilizó la analogía entre lo domesticado y lo silvestre como un recurso explicativo, también es cierto que la empleó como parte de un método de investigación científica en el que las analogías entre fenómenos permiten agruparlos si comparten la misma explicación causal a través de generalizaciones. Darwin empleó la metodología de la *vera causa* al establecer la selección natural como la causa responsable del cambio evolutivo, a partir de las generalizaciones que extrajo del proceso de selección artificial como un proceso completamente probado (Gildenhuys 2004, p.595; Wilner 2005, p.37; Theunissen 2012, p.181).

En *On the origin*, argumentó que la selección era el mecanismo que podía explicar la separación evidente entre linajes. En particular, la “selección metódica” permitía describir fácilmente cómo se lograba la modificación de una forma silvestre hasta un estado completamente domesticado, gracias a la acumulación diferencial de variaciones favorables en condiciones de un estricto control e intencionalidad humana. Posteriormente, definió la idea de “selección inconsciente” para sostener que esa acumulación desigual de rasgos podía escapar del auspicio e intervención del ser humano. Darwin encontró que, en la historia de la domesticación, la selección inconsciente había sido fundamental, ya que era inevitable que la selección natural actuara incluso en un ambiente creado por el hombre.

La selección inconsciente representaba un proceso lento, insensible y no teleológico, a diferencia de la selección metódica (Darwin, 1859, p.34-35). Extendió esta generalización destacando que el favorecimiento de algunas variaciones dentro de las poblaciones podía ocurrir sin intervención humana, con resultados semejantes, es decir, formando razas o incluso con efectos más potentes, hasta el punto de formar especies. El razonamiento de Darwin fue un ejercicio de generalización inductiva en el que la selección de variaciones heredables era suficiente para explicar la transformación de un tipo ancestral en variedades y especies (Gildenhuys, 2004, p.598-599).

Alter (2007, p.77) ha señalado que la analogía entre selección artificial y selección natural se fue modificando poco a poco desde sus textos tempranos (1842 y 1844), y fue hasta la publicación de 1859, y sus cinco ediciones posteriores, que esta idea se mantuvo prácticamente inalterada. Una década después, en la publicación de *Variation*, Darwin continúo reforzando la analogía con la copiosa evidencia que presentó y subrayó el papel de la selección artificial dentro de los diferentes modelos de manejo reproductivo (tales como hibridación, cruzamiento y entrecruzamiento), además de extrapolar su explicación hacia escenarios naturales, para integrar a la selección artificial como parte de un espectro en la historia evolutiva de las especies.

Antes del trabajo de Darwin, el principio de selección artificial fue entendido como un proceso por el cual la variación transmitida intergeneracionalmente conducía a la diferenciación de fenotipos, pero dentro de los límites propios de cada especie, concebida como una entidad fija. Difícilmente se habría interpretado como un mecanismo análogo al de la formación de nuevas entidades específicas e independientes del tipo ancestral. De hecho, esto fue considerado inicialmente como un punto débil muy importante en la propuesta de Darwin, no solo por sus oponentes antitransformistas. El propio Alfred R. Wallace, quien también propuso la teoría de la selección natural, juzgó inadmisibles las evidencias provenientes de la domesticación para respaldar los argumentos sobre la generación de nuevas especies (Richards, 1998, p.106; Bock, 2009, p.8; Varno, 2011, p.23).

En *Variation*, Darwin (1868) enfatizó en su narrativa el comienzo casi simultáneo de la selección metódica y la selección inconsciente. Particularmente, con la selección inconsciente, aunque se conservaban individuos que poseyeran características de interés, también se conservarían indirectamente otros rasgos (no necesariamente útiles) que, al heredarse, afectarían en alguna medida la diferenciación de las razas, sin que esto hubiese sido el propósito deliberado del criador (Darwin, 1868, p.212). Por esta razón, la selección inconsciente podía ser incluso más importante que la metódica, pues, al no tener un objetivo definido, la potencialidad en la modificación probablemente podría ser mayor que con la selección metódica. Al prescindir de una finalidad, esta noción podría cerrar la brecha entre la intervención metódica por parte del ser humano (selección artificial) y la naturaleza inmersa en el azar (selección natural) (Alter, 2007, p.60). Esta es una interpretación que permite entender a la selección natural y a la selección artificial, tanto metódica como inconsciente, como un modelo continuo más que como una dicotomía categórica (Gregory, 2009, p.6).

De acuerdo con Darwin, la selección artificial había generado la divergencia que dio origen a la razas y variedades modernas, al alejarse de su ancestro silvestre (Darwin, 1868, p.239). Aunque en el proceso de domesticación, esta separación de los linajes podría no ser tan intensa, sí implicaba el cambio en un subconjunto de individuos procedentes de una especie original y la causa principal eran los diferentes contextos selectivos. Para las formas silvestres, estas presiones están relacionadas con la supervivencia y la reproducción en la naturaleza mientras que, para las formas cultivadas, las presiones las imponen los domesticadores (Gregory, 2009, p.21). A partir de este razonamiento, tuvo sentido que Darwin concluyera que esta separación del tipo ancestral, a lo largo de una escala temporal más profunda y de contextos más complejos, podría explicar el origen de todas las especies.

## Los métodos de reproducción selectiva y su aplicación a la analogía selección artificial-selección natural

Aunque Darwin siempre otorgó mayor relevancia al principio de selección como la causa del mejoramiento de las razas (Theunissen, 2012; Del Savio, Mameli, 2020, p.21), también se interesó en explicar el papel de los métodos reproductivos para el desarrollo de las formas domesticadas (Derry, 2015, p.20; Bradshaw, 2016, p.35). Prácticas como la hibridación, el cruzamiento y el entrecruzamiento, las cuales involucraban no solo la gestión de los recursos disponibles (alimento, tipo de suelo, abonos, espacio, cercado, entre otros), sino también la selección de los “mejores” organismos, los más llamativos o exóticos, se utilizaron para favorecer su reproducción y en consecuencia replicar esas características en la descendencia.

A continuación, se discutirá la interpretación que Darwin (1868) desarrolló en torno a estas técnicas en el segundo volumen de *Variation*, ya que, al integrar estos conceptos a su analogía, intentó delinear escenarios aplicables a formas tanto domesticadas como silvestres.

### Hibridación

La hibridación, como una estrategia en el manejo de formas domesticadas, se basó en la reproducción entre individuos de diferentes especies. Sin embargo, el término “híbrido” llegó a emplearse para hacer referencia a un organismo que procediera de la cruza entre individuos de la misma raza/variedad o entre miembros de distintas razas, pero considerados de la misma especie (Wood, 2003, p.38).

Tanto en *On the origin* como en *Variation*, Darwin ocupó la palabra *mongrel* (mestizo) para referirse en particular a la descendencia obtenida con la cruza de variedades diferentes pero consideradas la misma especie, y el concepto *hybrid* para la cruza de especies distintas (Theunissen, 2012, p.185). Sin embargo, esta categorización, aunque útil en términos prácticos, fue discutida indirectamente por el propio Darwin, al sostener que los criterios (similitud morfológica, interfertilidad y esterilidad) que separan a las variedades de las especies no son parámetros exactos, sino que varían a lo largo de un espectro que se relaciona con la historia de vida de los individuos y con la historia evolutiva de la especie en cuestión.

No se comprendían con exactitud los mecanismos subyacentes a la replicación intermedia o mezclada de los rasgos en los individuos híbridos, aunque de acuerdo con la idea de herencia líquida, se reconocía de manera general que la hibridación daba como resultado plantas estériles con fenotipos intermedios (Müller-Wille, 2003, p.52). No obstante, numerosas evidencias empíricas y experimentales, proporcionadas por criadores y naturalistas, permitieron comprender que el fenómeno biológico de la hibridación era mucho más complejo que solo esperar descendencia estéril y morfologías intermedias.

En la primera edición de *On the origin*, Darwin (1859) dedicó un capítulo, titulado “Hybridism”, a discutir aquellos criterios a los que se apelaba con más frecuencia para establecer e identificar taxonómicamente la categoría de especie y que, en última instancia, se usaban para defender la inmutabilidad de los seres vivos: la compatibilidad reproductiva evidenciada en la interfertilidad y la esterilidad de los cruces híbridos. De acuerdo con Darwin, la esterilidad que se presentaba en los productos de los cruces híbridos no podía explicarse como un resultado de la selección natural, pues no sería ventajoso que los organismos perdieran la capacidad de dejar descendencia (Darwin, 1859, p.245). Sin embargo, el resultado de la hibridación no siempre era una descendencia completamente estéril, e incluso llegó a plantear que cuando se observaba esterilidad en organismos híbridos, probablemente se debía a la autofecundación que se promovía durante varias generaciones para replicar esa estirpe (p.248).

En *Variation*, Darwin (1868) revisó las investigaciones, tanto de naturalistas como de expertos en mejoramiento vegetal desde finales del siglo XVIII. La mayoría de sus discusiones sobre la hibridación no se modificaron en absoluto, pero en esta publicación se sumó una perspectiva relacionada con el tema de la variabilidad y sus posibles causas. Por ejemplo, señaló que, para algunos de los expertos, la hibridación representaba una fuente de variación inducida (al igual que el cruzamiento entre variedades) (Darwin, 1868, p.180), aunque subrayando que no se trataba de una causa última de este proceso.

De acuerdo con Darwin (1868), los patrones que se observaban en el cruzamiento, intra o intervarietal, eran los mismos que se podían esperar en la hibridación (excepto el fenómeno de la esterilidad-fertilidad). Por lo tanto, la reproducción sexual en general, ya fuese por cruzamiento o hibridación, representaba una fuente de variación en la descendencia mestiza o hibrida, respectivamente. Esta propuesta estaba directamente relacionada con el reconocido hecho de que las variedades naturales, las especies y las variedades domesticadas difieren solo en grado, no en tipo (Varno, 2011, p.26).

Al comparar los cruces entre variedades y entre especies, el propósito de Darwin fue señalar que los procesos que se observaban en cualquiera de estas categorías eran los mismos y, por lo tanto, los patrones esperados también eran semejantes. También argumentó que estos mismos procesos generales eran semejantes para mestizos e híbridos, al igual que para formas domesticadas y especies silvestres, para demostrar que las especies no son entidades estables, razón por la que no se podían aplicar criterios estrictos para identificarlas, pues ya fuese en estado natural o bajo domesticación, los organismos variaban inherentemente y se encontraban en contextos dinámicos y selectivos distintos, lo que ocasionaba la diferenciación y especialización de las poblaciones.

La hibridación fue un modelo que le permitió a Darwin cuestionar el razonamiento tradicional sobre la distinción teórica y metodológica entre especies y variedades. Gracias a los avances en la experimentación, se podía demostrar que las barreras reproductivas entre especies y variedades eran causadas por una ley de reproducción común a ambas categorías. En consecuencia, ni la esterilidad ni la fertilidad ofrecían un criterio consistente de la distinción entre especies y variedades (Wilkins, 2009, p.151; Varno, 2011, p.27). Ello explica que haya visto a las variedades como “especies incipientes” (Darwin, 1859, p.52, 1868, p.185-186), pues finalmente podían llegar a ser especies genuinas.

### Cruzamiento

El cruzamiento ha sido una técnica empleada por los domesticadores en la reproducción sexual de los organismos, en la que se controla y dirige el apareamiento entre individuos de la misma raza o variedad (Wood, 2003, p.30). Si podía asegurarse que dos individuos con similitud morfológica solo eran variedades de la misma especie, entonces era previsible que la reproducción fuese completamente posible, sin que se presentaran barreras de algún tipo.

Con el avance tecnológico de la época, diferentes asociaciones de criadores comenzaron una cercana colaboración con naturalistas, anatomistas y fisiólogos, para desarrollar explicaciones en torno a la variación, el desarrollo, la transmisión de rasgos y la modificación de razas, y en conjunto describieron los procesos generales subyacentes a tales fenómenos. Algunas de las primeras organizaciones fueron la Dishley Society, fundada en 1783, por Robert Bakewell junto con ganaderos la región central de Inglaterra, para el mejoramiento de razas ovinas (Wood, 2003, p.21), The Society for the Improvement of British Wool, originada en Edimburgo en 1791 (Poczai et al., 2022, p.6). Estas asociaciones pioneras, junto con la industrialización y el contexto beligerante entre territorios, estimularon el surgimiento de numerosas instituciones similares en estados de Europa continental, cuyo interés en la comprensión y manejo del proceso hereditario, fomentó una red de cultura material entre las poblaciones locales con intercambios al extranjero, no solo con un objetivo inmediato de comercialización (Wood, 2003; Wood, Orel, 2005; Poczai, Santiago-Blay, 2022; Poczai et al., 2022). Muchas de las explicaciones surgidas de la observación y experimentación con las formas domésticas frecuentemente eran extrapoladas a las especies naturales, más allá del control humano, para discutir su origen y cómo a través de la reproducción cruzada surgían modificaciones significativas (Poczai, Santiago-Blay, 2022, p.7).

Aunque domesticadores y naturalistas eran conscientes del surgimiento de variaciones espontáneas (reconocibles morfológicamente) en la constitución de los individuos (Wood, 2003, p.35), la fuente de variabilidad más evidente era el cruzamiento intervarietal. De hecho, la gran diversidad observada, al menos en las producciones domésticas, se atribuía a la cercanía con la que se cultivaban o criaban variedades o razas, pues esto facilitaba la reproducción cruzada (no siempre dirigida intencionalmente) (Oghina-Pavie, 2015, p.56). El cruzamiento fue considerado por Darwin como una fuente de variabilidad (Browne, 1980, p.66), al igual que la hibridación (Winter, 2000, p.431).

Darwin puntualizó que el cruzamiento producía dos patrones opuestos. Por un lado, podía evitar la formación de nuevas razas, produciendo homogeneidad en el conjunto de individuos, siempre y cuando estas cruzas se efectuaran entre individuos estrechamente relacionados. Por otro lado, también podía conducir a la modificación de las razas antiguas para generar nuevas variantes, cuando el cruzamiento se llevaba a cabo entre razas diferentes (Theunissen, 2012, p.195). Esta segunda tendencia estaba relacionada con la aportación de variación necesaria para la diferenciación de rasgos, así como con el aumento de vigor constitucional y de la fertilidad de la descendencia cruzada, lo que, de acuerdo con Darwin, contribuiría a que los mestizos se multiplicaran más rápidamente que las razas parentales puras (Darwin, 1868, p.85-86).

Ya que el cruzamiento podía introducir variación en los organismos domesticados, la mezcla de dos razas puras conducía a fenotipos intermedios, pero generación a generación se podían presentar rasgos mucho más variables (Wood, 2003, p.38). Esta variación inducida resultaba útil, pues los caracteres novedosos podían favorecer una futura selección metódica y, en consecuencia, generar nuevas razas (Wood, 2003, p.39; Poczai, Santiago-Blay, 2022, p.6). Este hecho fue aplicado por Darwin en su esquema, no solo en las formas domesticadas, sino también en las silvestres, pues el cruce libre entre individuos podía generar variaciones heredables que potencialmente otorgaran alguna ventaja en condiciones naturales (Darwin, 1868, p.90).

Es decir, Darwin continuó la defensa de la generalización del principio de selección, aplicando su argumento en un modelo donde el cruzamiento libre, en condiciones silvestres, también habría formado parte de la historia de las especies, proporcionando variación heredable y ventajosa, que contribuía a la separación de los linajes. Además, según la experiencia de los criadores, era común que entre las formas parentales que se cruzaban, generalmente una tuviese mayor capacidad para transmitir sus rasgos en la descendencia, es decir, tenía “prepotencia”. Asimismo, las razas autóctonas tenían una mejor adaptación a las condiciones locales, con respecto a formas introducidas (Wood, 2003). Para Darwin (1868, p.89), esta evidencia demostraba que la selección natural no era suprimida incluso en condiciones de domesticación.

### Entrecruzamiento cercano

El cruzamiento de individuos estrechamente relacionados, no solo pertenecientes a la misma variedad, sino con un estrecho lazo consanguíneo, se volvió una práctica común en la gestión de los organismos domesticados desde finales del siglo XVIII y se intensificó durante el siglo XIX (Orel, Wood, 1981; Wood, 2003; Derry, 2015; Poczai, Santiago-Blay, 2021). En animales, la técnica consistía en la selección de los organismos que presentaban los rasgos de interés y al igual que en un cruzamiento común, dirigir el apareamiento para la procreación de descendencia, solo que los individuos elegidos debían tener una relación genealógica muy cercana (padre e hija, madre e hijo, hermano y hermana etc.). En plantas, la técnica era fundamentalmente la misma. Los ejemplares más estrechamente emparentados podían ser cruzados, aunque en este tipo de organismos, la proximidad podía llegar hasta el grado de la autofecundación.

Como se mencionó previamente, además de añadir variabilidad, el cruzamiento también permitía la replicación fiel de los rasgos típicos en una raza, mediante la reproducción de organismos cercanamente emparentados, es decir, el entrecruzamiento, el cual permitía la conservación de los caracteres y la restricción de variación. El método conocido como *breeding in-and-in* fue desarrollado por el experto en cría de ovejas Robert Bakewell, pionero en la implementación de este sistema, como parte de un modelo de producción masiva, el cual se aplicó al mejoramiento de diferentes ganados. La estrategia combinó la selección cuidadosa de individuos vigorosos, fecundos y con caracteres notables, seguido de la reproducción entre consanguíneos (Orel, Wood, 1981, p.153-154).

Esta técnica se volvió indispensable en la cría intensiva porque mediante este procedimiento se obtenían razas homogéneas o puras (Wood, 2003, p.32; Stoykovich, 2010, p.36; Poczai, Santiago-Blay, 2021, p.5). Es decir, en la historia de aquellas razas bien caracterizadas, cuya importancia económica era elevada, era necesaria una etapa de entrecruzamiento cercano para la estandarización del fenotipo asociado a ese valor estético y/o económico.

Desde *On the origin*, Darwin había señalado que el entrecruzamiento cercano traía como consecuencia una disminución en el vigor constitucional de la descendencia, así como esterilidad. Sin embargo, no fue un tema que haya profundizado más allá de la mención de testimonios generales de expertos en cría animal. Fue en *Variation* donde amplió la discusión sobre estas prácticas optimizadas por los domesticadores de la época, señalando que el entrecruzamiento permitía la observación de varios fenómenos biológicos en los organismos domesticados, que sin problema podían extrapolarse a las poblaciones naturales, por lo que se trataba de un proceso que probablemente pudo tener un papel relevante en la historia de las especies en general, no sólo de las domesticadas.

En condiciones silvestres, Darwin (1868, p.114-115) explicó que la persistencia del entrecruzamiento en una población se relacionaba con aspectos tales como una baja densidad poblacional, poca probabilidad de hibridación y una distribución espacial restringida. Para explicar su punto, tomó como ejemplo una comparación entre ganado salvaje británico (confinado en un área privada) y ganado semi-salvaje de Paraguay (con menor control del cruzamiento libre), con lo cual intentaba demostrar que la fertilidad se ve disminuida en aquellas poblaciones donde el entrecruzamiento cercano había ocurrido a lo largo de las generaciones, por lo menos, hasta donde los registros permitían conocer (p.119).

En los organismos domesticados, la reproducción consanguínea era el medio para obtener razas puras mejoradas, pero generalmente se asociaban con esta práctica la pérdida de fertilidad, la presencia de malformaciones o capacidades deterioradas (Wood, 2003, p.31; Poczai, Santiago-Blay, 2021, p.6-7). Este fue el caso del ganado vacuno Shorthorn, originario del noreste de Inglaterra, el cual había sido entrecruzado extensivamente desde finales del siglo XVIII y hacia la mitad del siglo XIX, que presentaba más crías con malformaciones, en comparación con otras razas (Darwin, 1868, p.118).

Sin embargo, el tiempo que se requería para manifestar los efectos adversos a veces podía ser prolongado, ya que la acumulación de rasgos perjudiciales era gradual. Darwin (1868, p.115-116) mencionó que durante la primera mitad del siglo XIX se había intensificado esta práctica y que una medida precautoria tomada por algunos expertos consistió en moderar el uso del entrecruzamiento entre parientes cercanos y favorecer cruzas ocasionales con individuos de la misma raza, pero no con una estrecha relación genealógica, ya que era una técnica probada para dotar de vigor a los descendientes.

Una vez que se lograba “esculpir” la morfología deseada, la reproducción consanguínea permitía alcanzar cierta estabilidad hereditaria, al impedir, tanto como fuera posible, que surgieran variaciones que se desviaran de esa tipología. No obstante, los criadores eran conscientes de la aparición constante de características fortuitas, así como de los efectos adversos propios de la consanguinidad, y comprendían que, en cierta medida, estas consecuencias no deseadas escapaban a todo control selectivo, pues, aunque un individuo no mostrara un defecto, no se podía asumir que no lo portaba y que existía la posibilidad de heredar esa desventaja (Wood, Orel, 2005, p.245).

La causa de la degeneración e infertilidad ocasionadas por la consanguinidad no se comprendió con exactitud hasta el desarrollo de la herencia mendeliana, como un área de investigación en el siglo XX. Sin embargo, se llegó a conjeturar que, en el entrecruzamiento, era más probable que los individuos estrechamente emparentados que se reproducían poseyeran por ascendencia las mismas variaciones perjudiciales que se intensificaban en su descendencia, mientras que, en el cruzamiento de razas diferentes, los progenitores tenían variaciones distintas que podrían compensarse en su progenie (Zirkle, 1952, p.11).

## El significado de domesticación en *Variation*

Hacia la segunda mitad del siglo XVIII, se desarrollaron conceptos nuevos, estimulados por la creciente confianza en la intervención humana sobre la naturaleza, lo que implicó una interpretación del proceso de domesticación como la dominación del mundo natural por parte del ser humano. Las prácticas de cría y cultivo desarrolladas y perfeccionadas entre el siglo XVIII y XIX reforzaron estas nociones, lo que llevó a visualizar a la domesticación como la gestión y sistematización del manejo de los organismos para su mejoramiento, bajo criterios y estándares específicos. Hacia la segunda mitad del siglo XIX, la palabra “domesticación” comenzó a emplearse dentro de la jerga científica y económica en espacios especializados como algunas sociedades de aclimatación, en donde el criterio definitorio fue la reproducción bajo el control humano (Bogaard et al., 2021, p.5), un parámetro que ha prevalecido en múltiples definiciones modernas.

Aunque las formas domesticadas habían sido problematizadas en el marco del debate transformista, desde principios del siglo XIX, no había existido una conceptualización manifiesta del término “domesticación” dentro de ese nuevo contexto. Las similitudes con sus contrapartes silvestres eran evidentes, lo que en algunos casos permitía concebir un origen común (entre domesticado y salvaje de la misma especie), pero la explicación de cómo se habían diferenciado, desde hacía cuánto y en dónde, o si la potencialidad para el cambio era un atributo exclusivo de las formas domesticadas, o más bien un atributo general de todas las especies, fueron cuestiones que se abordaban de manera aislada.

Para Darwin, el concepto “domesticación” significaba un proceso experimental de transformación en el que los organismos se adaptan a un entorno fabricado por el ser humano. La transición de lo salvaje a lo domesticado, en relación con la escala geológica, podría no evidenciar un cambio tan pronunciado que permitiera asegurar el surgimiento de una nueva especie. Sin embargo, en una escala temporal que se acotara a algunas generaciones, las modificaciones efectivamente serían significativas. No solo tomando en cuenta cambios en el comportamiento de animales, o alteraciones evidentes en la apariencia de los organismos. La domesticación se habría logrado una vez que ciertos atributos como fecundidad, variabilidad y plasticidad se lograran sin esfuerzo o incluso se hubiesen incrementado, lo que demostraría un ajuste a las condiciones de vida modificadas (Darwin, 1868, p.405). Tales transformaciones, al contrastarse con la tipología de las formas silvestres, evidenciaban una separación histórica de los linajes, que se replicaba generación a generación, lo que reforzaba una separación reproductiva y que, además, dicho proceso podía encaminarse en numerosas direcciones, obedeciendo a diferentes circunstancias.

Asa Gray, uno de los corresponsales más importantes de Darwin en EEUU, defendió la importancia de la proposición darwiniana sobre las variedades como especies incipientes. Esto permitió considerar y observar detenidamente a los organismos domesticados y lo que podían probar sobre la derivación de las formas, ya que al poder convertirlos en algún tipo de “monstruosidad”, aunque no se podrían originar ni perpetuar en la naturaleza, no dejaban duda del poder ilimitado de la variación, así como de la universalidad de esa tendencia, no solo en los domesticados, sino en los organismos silvestres, aunque con particularidades que se relacionaban con su entorno y con su historia específica (Gray, 1876, p.26).

Por su parte, el concepto de “selección artificial”, que Darwin instrumentalizó para respaldar su teoría, hacía referencia a un proceso de acumulación de rasgos mediante la intervención humana (intencionalmente o no) en la reproducción de los organismos, para generar divergencia en los caracteres. Los atributos en cuestión tendrían que ver con la mansedumbre en el caso de los animales, la fecundidad, la persistencia de rasgos juveniles en la etapa adulta, o bien, en las plantas, un crecimiento rápido y ciclo de vida corto, tolerancia a diferentes condiciones climáticas, producción de semillas grandes o fáciles de cosechar, entre otros. La separación y replicación de estas características, es decir mediante la selección de los organismos que podrían reproducirse, generaría diferenciación y especialización en la morfología. Lo anterior podría ocurrir esencialmente, tanto en la selección natural como en cualquier categoría de la selección artificial.

Para Darwin, el factor causal para la ocurrencia de domesticación, así como de la transformación natural, era el principio general de selección. La visión que presentó en el primer capítulo de *On the origin* y que extendió en *Variation* sostuvo que la domesticación demostraba en sí misma la ocurrencia de cambios (fenotípicos, hereditarios y ecológicos) en las subpoblaciones que experimentaban procesos vinculados al manejo humano. La generalización de estos mecanismos le permitió establecer las afinidades entre la domesticación y el proceso evolutivo, ya que, en ambas, el elemento causal más poderoso era el principio de selección (Gildenhuys, 2004, p.596). A partir de este marco teórico desarrollado por Darwin, la domesticación y la selección artificial comenzarían a entenderse como procesos evolutivos muy específicos, adquiriendo una nueva acepción semántica.

Para ello delineó una narrativa en la que describió la domesticación como un proceso histórico que involucraba diferentes etapas con distintos mecanismos clave en cada episodio. Fue así que concatenó diferentes componentes (variación, herencia y reproducción) necesarios para explicar la selección inconsciente y metódica, efectuada a través de las estrategias de reproducción selectiva (hibridación, cruzamiento y entrecruzamiento) para establecer estas posibles etapas en la historia de una especie domesticada. El inicio de la domesticación necesariamente tuvo que partir de un conjunto de individuos silvestres, ya fuesen miembros de la misma especie o productos de una hibridación previa (lo que habría respaldado que la hibridación ocurre en condiciones silvestres previas al manejo humano). En esa etapa inicial, el cruce interespecífico (hibridación) o intraespecífico (cruzamiento) aportaría variación y vigor constitucional, atributos que serían valiosos para el ser humano. A partir de ejemplares híbridos o mestizos, se podrían seleccionar los fenotipos de mayor interés y generar diferentes razas mediante cruces intervarietales. Finalmente, la homogenización de una raza se lograría restringiendo la variación, empleando el entrecruzamiento y aplicándolo una generación tras otra, teniendo cuidado en seleccionar siempre los mejores ejemplares.

Estos procesos estaban registrados en las prácticas antiguas de cría y cultivo, pero en condiciones silvestres no existían registros abundantes. A pesar de ello, Darwin investigó y consideró algunos ejemplos de poblaciones salvajes o semi-salvajes, en los que existía evidencia de hibridación, cruzamiento o entrecruzamiento. Además, señaló en qué circunstancias podrían llegar a ocurrir, ya fuese aislamiento geográfico como resultado de alteraciones geológicas, migraciones o por la colonización de nuevos espacios, aunque siempre ponderando el principio de la selección como la causa fundamental de la diferenciación. Es decir, en la historia de una especie que fue domesticada o en la historia de una especie transformada en la naturaleza, los procesos fundamentales para su modificación eran los mismos.

El enfoque de Darwin resultó novedoso al integrar diferentes elementos, a veces aparentemente inconexos, como la variación, la reproducción junto con la herencia y la adaptación al entorno, en una explicación generalizadora: los organismos silvestres atraviesan por procesos de selección natural mientras que los domesticados pueden pasar por selección natural y artificial incluso simultáneamente. Ello genera cambios observables y medibles en los organismos, lo cual conduce a la diferenciación de las poblaciones (razas y especies) (Darwin, 1868, p.409-410).

La resignificación que Darwin planteó sobre la domesticación y la selección artificial, así como la forma en que integró estas nociones a su esquema, tuvo implicaciones importantes para las diversas interpretaciones que se harían de su teoría. Tanto opositores como seguidores de Darwin vieron la analogía entre domesticación y evolución como un planteamiento contradictorio y como una prueba incompatible con la conclusión que él quería defender (Richards, 1998; Gregory, 2009, p.7). Sin embargo, la evidencia que se recuperaba de los modelos domesticados respaldaba las interpretaciones darwinianas, y, en consecuencia, la domesticación sirvió para demostrar su visión y convencer a algunos contemporáneos, como Joseph D. Hooker (Bellon, 2006, p.31) y Alphonse de Candolle (Alvarez-Tostado et al., 2024, p.463).

Lógicamente, Darwin siempre defendió la relevancia de su propuesta. No obstante, otros mecanismos subyacentes equiparables a la selección artificial como promotoras de cambio (no adaptativo) escaparon a su análisis. Estuvo de acuerdo con un escenario inicial de domesticación, en el que los humanos comenzaron a aislar los recursos administrados de las poblaciones de vida libre, especialmente cuando fueron trasladados fuera del área de distribución natural de sus progenitores. Ahora se sabe que intervienen otros procesos. Además de la selección artificial, entran en juego mecanismos como la deriva génica y el flujo génico (no solo en el comienzo de la domesticación, sino recurrentemente a lo largo de la historia de una especie domesticada) (Gregory, 2009; Allaby, Ware, Kistler, 2018; Moreno-Letelier et al., 2020). De hecho, algunos enfoques modernos consideran que la selección artificial a través de la cría deliberada para fomentar rasgos específicos es un desarrollo relativamente tardío en la mayoría de las especies domesticadas (Zeder, 2012; Zeller, Göttert, 2019).

## Consideraciones finales

A partir de la publicación de *On The origin*, parece que Darwin se comprometió con la labor de defender la importancia científica del principio de selección. En el pensamiento que desarrolló en la obra *Variation*, tuvo como objetivo reforzar la causalidad de la selección natural, señalando en su esquema la causalidad comprobada de la selección artificial sobre la tendencia a la diferenciación entre razas, de acuerdo con los contextos de cría y cultivo. Al demostrar la divergencia de rasgos que se generaba entre subpoblaciones, a partir de la cría selectiva, fue posible afirmar que la selección natural es una fuerza de mayor orden, con alcances más definitivos, como la transformación de las especies.

En la perspectiva de Darwin sobre la domesticación estaba implícito el control del ser humano y el manejo de las especies como recursos aprovechables, no solo al concebirlas como ganado, cultivos, fuentes materiales e incluso estéticas. Los procesos biológicos también representaban una forma de recurso, como la capacidad natural de variar, la capacidad de heredar rasgos, la capacidad de reproducir tipos morfológicos, la capacidad de ajustarse a nuevos entornos o la capacidad de ser modificables. Estos mecanismos, que durante mucho tiempo fueron misteriosos y difíciles de manipular, eran la materia prima para que la domesticación ocurriera. Considerar la universalidad histórica de estos componentes probablemente facilitó que Darwin concibiera la domesticación como un tipo particular de escenario evolutivo, restringido temporalmente, en el que los organismos se adaptan a un entorno fabricado por el ser humano.

Antes de la consolidación del pensamiento evolucionista, la gestión de los recursos domesticados implicaba la intervención y el manejo en la reproducción de los individuos. Los métodos que se aplicaban para tales fines incluyeron la hibridación, el cruzamiento y el entrecruzamiento cercano. Cada uno permitía un manejo eficiente de los recursos, dependiendo del objetivo que se perseguía. La hibridación permitía introducir variación interespecífica, mezclar caracteres de linajes distintos e incluso se llegó a suponer que podían crearse especies completamente nuevas. El cruzamiento también representaba variación, aunque dentro de límites más estrechos, generando casi invariablemente individuos interfértiles, lo que permitía la replicación iterativa para la producción de razas o variedades. El entrecruzamiento cercano se encontraba al otro extremo de la hibridación y permitía la homogeneidad en los individuos domesticados, lo que prácticamente garantizaba una producción constante de los rasgos de interés.

La noción de domesticación, la idea general de selección y las estrategias de manejo reproductivo ya gozaban de importancia entre los conocimientos de los domesticadores y naturalistas, incluso sin que existiera la noción evolucionista. Sin embargo, adquirieron una completa resignificación al ser interpretados a la luz de la teoría darwiniana. En la actualidad se acepta que tanto la domesticación (modificación y adaptación a entornos mediados por el ser humano) como la selección artificial (acumulación de variaciones particulares mediante el control reproductivo) describen procesos evolutivos en sí mismos.

## Data Availability

No están en repositorio.
